# The Political Salience of Animal Protection in the Republic of Ireland (2011–2020): What Do Irish Political Parties Pledge on Animal Welfare and Wildlife Conservation?

**DOI:** 10.3390/ani14243619

**Published:** 2024-12-15

**Authors:** Annick Hus, Steven P. McCulloch

**Affiliations:** 1 Edinburgh Medical School, Biomedical Sciences, Hugh Robson Building, Edinburgh EH8 9XD, UK; 2 Centre for Animal Welfare, University of Winchester, Winchester SO22 4NR, UK; steven.mcculloch@winchester.ac.uk

**Keywords:** animal protection, animal welfare, conservation, issue salience, party politicisation, Republic of Ireland

## Abstract

Ireland is the largest beef trader and a major dairy exporter within the European Union. Horse racing holds significant cultural value, while greyhound racing is economically important at the regional level. As public concern about farm animal welfare rises and biodiversity loss intensifies, this research examines the political salience of animal protection in Ireland across the 2011, 2016, and 2020 general elections. Political salience measures the degree of public concern about political issues, with party manifestos serving as indicators of this concern. Using both quantitative and qualitative methods, this study analysed 364 statements on animal protection from Fianna Fáil, Fine Gael, the Green Party, the Labour Party, and Sinn Féin. The results show a significant increase in the frequency and positivity of animal protection statements over the course of the election period, with a shift in the attitudes of Fianna Fáil and Fine Gael. The Green Party has consistently promoted progressive animal protection policies. Key issues such as farmed animal welfare, wildlife and biodiversity, and horse and greyhound racing have gained prominence in party manifestos, also reflecting economic influences. Despite this growing focus, critical issues such as the puppy trade and certain aspects of farmed animal welfare remain underrepresented. This research provides the first comprehensive academic analysis of animal protection policy in political party manifestos in the Republic of Ireland.

## Simple Summary

Ireland’s political engagement with animal protection has evolved significantly between 2011 and 2020, reflecting growing societal concerns about animal welfare and biodiversity. This study analysed election manifestos from Ireland’s main political parties during this period, examining how these issues have gained prominence. Key topics include farmed animal welfare, wildlife conservation, and the economic and ethical dimensions of horse and greyhound racing. While progressive parties such as the Greens consistently advocated for strong animal welfare policies, centrist parties such as Fianna Fáil and Fine Gael gradually adopted more positive stances, albeit often with an economic focus. Despite this progress, critical issues such as the puppy trade, livestock welfare, and sustainable farming practices remain underrepresented. This study highlights a growing public and political recognition of the importance of animal welfare, with implications for future policy development. As awareness increases, there is potential for more robust legislative action to address gaps and strengthen protections for animals and ecosystems. This research, the first of its kind in Ireland, sheds light on the political dynamics surrounding animal welfare and their alignment with societal values. It provides a foundation for understanding how public sentiment influences policy and underscores the importance of continued advocacy and research in this field.

## 1. Introduction

This research examines the political salience of animal protection in the Republic of Ireland by analysing the election manifestos from the 2011, 2016 and 2020 general elections. Growing scientific evidence of animal sentience and cognition has highlighted the ethical imperative to address animal suffering [1,2]. These findings, coupled with shifting societal attitudes and advocacy efforts led by non-governmental organisations, have driven significant advances in public policy and legislation, such as the EU Directive 2010/63/EU on animal welfare [3,4,5].

Ireland, with a population of approximately 5.15 million [6], occupies four-fifths of the island of Ireland and has been a member of the EU since 1973. It has a GDP of EUR 480 billion [7]. With around 100,000 production farms and a herd of 6.6 million cattle, beef farming plays an important role in Irish agriculture, accounting for over 25% of the total agricultural output. There are 889,000 suckler cows, making Ireland’s herd the third largest in the EU. With 1.5 million dairy cows, the country is also a major dairy exporter [8,9]. Ireland maintains 2 million pigs, including 145,000 breeding sows and 2 million laying hens and produces 70 million broiler chickens per year [10,11]. Recent welfare concerns have focused on dairy cows, calves, beef cattle, broiler chickens, and sows [12]. The *EU Animal Welfare Barometer* 2023 shows that 71% of the Irish population prioritises decent living conditions for farm animals, and 78% strongly supports sufficient space for movement [13].

Ireland has a diverse range of habitats supporting significant populations of birds, fish, mammals, invertebrates, plants, and fungi. However, 85% of EU-protected habitats are in unfavourable conditions, with 46% in decline, particularly in marine, peatland, grassland, and woodland areas [14]. Livestock production has exacerbated problems of overexploitation, pollution, and biodiversity loss [15]. While 90% of the population supports an improvement in biodiversity, 56% are unsure of how they can contribute [16].

Ireland’s horse and greyhound racing industry is recognised as a valuable economic contributor. According to Horse Racing Ireland [17], the horse racing industry employs approximately 30,000 people and contributes EUR 2.46 billion to the economy. An estimated 10,000 people benefit economically from the greyhound sector [18]. Both industries receive financial support from the Irish Government through the Horse and Greyhound Racing Fund of the Horse and Greyhound Racing Act 2001, which is considered vital to the survival and development of both horse and greyhound racing. Yet, both industries have come under increasing criticism, as highlighted by the documentaries The Dark Side of Horse Racing and RTÉ Investigates: Greyhounds Running for Their Lives, which exposed inhumane treatment and welfare issues [19,20]. Concerns have also been raised about Ireland’s large puppy breeding industry [21].

This research examines the political salience of animal protection (In this paper, the term ‘animal protection’ is used as an umbrella term to cover animal welfare, wildlife conservation, and animal rights.) in Ireland, a country with a multi-party system where proportional representation determines the composition of government. Political salience refers to the importance of a political issue to voters and its influence on their voting behaviour [22]. Election manifestos, which are designed to maximise electoral success, serve as a measure of the issues prioritised by voters and reflect the positions of political parties at particular moments in time [23,24].

This paper explores the following research questions:

1. How has the prominence of animal protection issues in Ireland evolved according to manifesto analysis of the 2011, 2016, and 2020 general elections?
2. Which animal protection issues have been most politically significant in Ireland from 2011 to 2020?
3. What is the relationship between the political orientation of Irish parties and their animal protection policies as outlined in their manifestos?

The following sections provide a review of the academic literature on salience in animal protection and the political context in Ireland. This paper then moves on to provide an overview of animal protection in Ireland, focusing on farmed animal welfare, horse and greyhound racing in Ireland, puppy farming, and wildlife and biodiversity.

## 2. Salience in Animal Protection

Several academic studies have examined the attention given to animal protection by political parties across different countries and time periods. Chaney [24] analysed the UK political landscape from 1979 to 2010, noting a gradual increase in attention to animal protection issues, with left-wing parties adopting more progressive policy positions than right-wing counterparts. Chaney et al. [25] conducted a detailed examination of Scotland, Wales, and Northern Ireland (animal health and welfare is a devolved responsibility in the UK) from 1998 to 2017, noting a rising prominence of animal welfare issues within electoral agendas across various levels of government.

Vogeler [26] performed a systematic review of farm animal welfare policies in Austria, Germany, and Switzerland, revealing distinct approaches across these countries. Vogeler [27] also explored the German political landscape, noting the prominent advocacy role of the Green Party in supporting animal welfare. Additionally, Vogeler [28] highlighted how societal concerns influenced political parties in Germany and the UK, leading to stricter regulations compared to the EU’s standards.

Hus and McCulloch [29] examined the political salience of animal protection by analysing party manifestos in the Netherlands and Belgium from 2010 to 2021. They found that animal protection gained in importance over successive electoral cycles. Left-wing parties adopted more progressive positions, prioritising animal protection over economic concerns, while right-wing parties focused more on economic interests. The study also highlighted that different levels of electoral politics offer opportunities to advance animal protection agendas.

Issue salience, a key concept in political science research, describes the relative importance and relevance of different policy issues [30]. As a metric for measuring the level of contention between political factions, issue salience highlights the extent to which individual parties attach importance to particular policy areas, and party manifestos provide repositories of this information at particular points in time. Despite the potential shortcomings associated with their use as data repositories, party manifestos can be invaluable resources for longitudinal research, particularly because they have tended to become more comprehensive documents in recent decades [24].

## 3. The Political Context in Ireland

The Republic of Ireland is a parliamentary republic with proportional representation and a bicameral parliament: the Dáil Éireann (lower house) and Seanad Éireann (upper house) [31]. Simon Harris has been Taoiseach (Prime Minister) since April 2024. The three largest parties are Fianna Fáil, Sinn Féin, and Fine Gael. Table 1 lists the Irish political parties that published manifestos for the 2011, 2016, and 2020 general elections analysed in this research.

In 2020, Fianna Fáil won 38 seats and formed a government with Fine Gael (35 seats) and the Green Party (12 seats). This Parliament marked the first power-sharing between Fianna Fáil and Fine Gael and the first government entry for the Green Party [32].

Fianna Fáil, established in 1926, dominated Irish politics after the Second World War but experienced a decline in seats in 2011 [33]. Alongside Fine Gael, established in 1933, Fianna Fáil’s manifestos supported high animal welfare standards but also endorsed horse and greyhound racing, live livestock exports, and wildlife protection [34,35]. The Green Party, established in 1981, increased its seats from 3 to 12 in 2020 [32] and advocated for more animal welfare regulations and the recognition of animals as sentient beings [36].

Sinn Féin, a left-wing republican party founded in 1905, emphasised animal protection in the context of economic interests related to farming and fishing [37]. It won 37 seats in 2020, but Fianna Fáil and Fine Gael refused to form a coalition, citing tax policy and links to the IRA [38]. The Irish Labour Party, founded in 1912, remains linked to the trade union movement and defends workers’ interests. In 2020, it pledged to tighten dog breeding laws and protect fish stocks after Brexit [38,39].

## 4. Animal Protection in Ireland

Farming, husbandry, companion animals, and sporting activities with animals are all an important part of contemporary Irish life. In 2021, the Irish Government published its Animal Welfare Strategy 2021–2025 to provide a coherent and ambitious approach to animal welfare based on five principles: facilitating respectful dialogue, enhancing cooperation, increasing capacity, improving coordination, and improving animal welfare in multiple situations [40].

### 4.1. Farm Animal Welfare

#### 4.1.1. Beef Farming

Ireland’s livestock industry includes 6.6 million cattle on 100,000 farms, making it one of the world’s top ten beef exporters. In 2020, 90% of its 524,543 tonnes of beef was exported, mainly to the UK and EU [9]. Effective management and appropriate housing are critical to cattle welfare, particularly in winter or when pasture is limited [41]. Farmer involvement improves welfare outcomes, including reduced lameness, less disease, and improved environmental and social conditions [42].

#### 4.1.2. Dairy Farming

Ireland has 1.5 million dairy cows on 17,000 farms [9], with dairy exports valued at EUR 6.3 billion in 2023 [43]. The Food Harvest 2020 programme and the removal of milk quotas spurred sector growth [44], raising its public profile [45]. In 2023, the industry faced criticism after RTÉ footage revealed the mistreatment of calves [46].

#### 4.1.3. Pig Farming

In 2022, Ireland had 1,634,800 pigs, including 1,497,600 non-breeding and 137,200 breeding pigs [47]. Most sows are kept in stalls for one month of gestation and then moved to farrowing crates [48]. The EU partially bans sow stalls, allowing their use only during early pregnancy and the week before farrowing. The European Food Safety Authority discourages stalls that restrict movement [49]. McCulloch [50] argues that farrowing crates should be banned as they do not meet sows’ welfare needs.

#### 4.1.4. Broiler Chickens

Irish farmers rear 110 million broilers annually [51]. In 2022, poultry meat exports surpassed 100,000 tonnes, valued at EUR 314 million [52]. Intensive farming practices, including mutilations, confinement, high stocking densities, and accelerated growth, lead to welfare problems such as disease, exhaustion, lameness, and heart failure in fast-growing broilers [53,54].

#### 4.1.5. Egg-Laying Hens

Ireland has 1.8 million caged laying hens, making up 54% of the national flock [55]. While the EU banned barren battery cages in 2012, it still permits larger modified cages with some enrichment, though they limit behaviours like nesting, dust bathing, and foraging [56]. The ISPCA and other NGOs advocate phasing out all cages by 2025 [48].

#### 4.1.6. Sheep Farming

Ireland has a sheep population of 4 million, with nearly 3 million slaughtered in 2022. It is the fourth largest global exporter and second in the EU, with exports valued at EUR 476 million [57]. Sheep transported on roll-on-roll-off ferries face welfare issues such as prolonged journeys, weather disruptions, and inadequate ventilation [58]. Other concerns include lameness, castration, tail docking, handling stress, and parasites [59]. In 2022, Ireland exported 15,000 live sheep worth EUR 2 million, mainly to Europe and the Middle East [60]. Live transport carries welfare risks, including stress and disease transmission [61].

#### 4.1.7. Aquaculture and Seafood Industry

Ireland’s fishing industry is valued at EUR 1.3 billion, with 1993 vessels and 296 aquaculture sites. Aquaculture, mainly salmon (EUR 119 million) and cod (EUR 44 million), is worth EUR 196 million. Its growth has raised concerns about biodiversity, pollution, antibiotic overuse [62], and animal welfare issues like overcrowding and poor water quality [63,64]. The seafood industry exports EUR 703 million, primarily to the EU (EUR 407 million), the UK (EUR 80 million), and Asia (EUR 79 million) [65]. Welfare concerns in UK marine fisheries include overcrowding and suffocation at slaughter [66].

### 4.2. Horse and Greyhound Racing

#### 4.2.1. Horse Racing

Established in 1994 to boost horse racing investment, the Irish Horse Authority was succeeded by Horse Racing Ireland (HRI) in 2001 as a quasi-governmental body to reinforce Ireland’s racing tradition [67]. In 2022, the Irish horseracing industry contributed over EUR 550 million in foreign direct investment and aims to expand its global market, enhance domestic impact, and lead in equine welfare and sustainability [17]. Despite the Irish Thoroughbred Welfare Council’s advocacy since 2000, a 2021 BBC investigation reported the slaughter of many young, retired, and predominantly Irish-bred racehorses [68]. Similar concerns were raised by the Irish Times about the treatment of exported racehorses [20].

#### 4.2.2. Greyhound Racing

Greyhound racing receives public funding of EUR 20 million [69]. Despite pandemic-related challenges, the industry showed resilience in 2021, recording an operating surplus of EUR 3.8 million from 1384 meetings with 91,778 greyhounds [70]. Yet, the documentary *RTÉ Investigates: Greyhounds Running for Their Lives* exposed welfare issues, including the annual euthanasia of 6000 greyhounds due to poor performance [19]. This ‘wastage’ [71] (p.3) and associated concerns about breeding and welfare conditions have been extensively documented [71,72]. Legislative efforts such as the *Greyhound Racing Act 2019* and the *Horse and Greyhound Racing Fund Regulations 2023* have sought to improve welfare. Animal welfare groups want to see the sport phased out within the next few years [18,73].

### 4.3. Puppy Farming

In Ireland, the number of registered dog breeding establishments surged from 73 in 2016 to 258 in 2018, leading to an annual production capacity of about 30,000 puppies [21]. This growth, particularly in large-scale operations with up to 500 breeding animals, raised animal welfare concerns [21]. In response, the Animal Health and Welfare (Sale or Supply of Pet) Regulations 2019 imposed stricter registration requirements for breeders of six or more dogs annually [74]. Despite these measures, Ireland faces a severe dog welfare crisis, with a 77% increase in dogs entering pounds in 2022, doubled euthanasia rates, and a notable rise in rehoming requests and rescues in 2023 [75].

### 4.4. Wildlife and Biodiversity

Ireland’s National Parks and Wildlife Service (NPWS), part of the Department of Housing, Local Government, and Heritage, oversees the protection and conservation of the country’s natural heritage. This includes designating Special Areas of Conservation (SAC), Special Protection Areas (SPA), and Natural Heritage Areas (NHA) and managing a licensing system to regulate activities impacting habitats and species [76]. The fourth National Biodiversity Action Plan (NBAP) for 2023–2030 aims to combat biodiversity loss, and the Wildlife (Amendment) Act 2023 introduces a biodiversity duty for public bodies [14]. Ireland supported the Nature Restoration Law in 2024 as part of the European Green Deal [77]. However, conservation challenges remain, with endangered bird species and underfunded nature conservation (the National Biodiversity Data Centre, 2022). The seafood industry exacerbates biodiversity loss through overfishing and aquaculture [62].

## 5. Methodology

### 5.1. Research Design

This research analyzes the election manifestos from the Irish national elections of 2011, 2016, and 2020, following the methodology of Hus and McCulloch [29]. Only manifestos from parties that participated in all three elections were considered (see Table 1). These manifestos, available in English and partially in Irish, were sourced from the Manifesto Project website (https://manifesto-project.wzb.eu/ (accessed on 3 June 2021)) and https://pidgeon.ie/manifestos/ (accessed on 3 June 2021).

A quantitative analysis was conducted using keywords such as animal(s), agriculture, fish(eries), biodiversity, slaughter, transport, and wildlife. Manifesto texts were segmented into quasi-sentences—statements expressing political ideas— and coded based on animal protection issues, similar to the method used by Chaney et al. [25]. Statements were categorised as pro-, anti-animal protection, or neutral, following Reingold’s [78] directional approach to analysing political debates. This method, combined with qualitative analysis, helps reveal party positions and political dynamics [24]. The categories for analysis are detailed in Table 2.

Only the statements directly related to animals were tagged, with multiple references to the same statement counted once. Statements covering multiple issues were tagged under the most relevant issue. The first author conducted the analysis of the manifestos twice.

### 5.2. Limitations

The methodology, based on Hus and McCulloch [29], employs a single-coding approach where each statement is tagged only once. This enhances the reliability of comparing statement counts across election years, allowing for a robust quantitative measure of ‘salience’. While tagging statements addressing multiple issues can be challenging, clear definitions for the issue categories (see Table 2) were established to ensure consistency. Additionally, to account for variations in the manifesto sizes and prevent misleading interpretations of absolute statement counts, the analysis incorporated the number of pages in each manifesto. These methodological steps strengthen the reliability and comparability of the findings, effectively addressing potential weaknesses.

## 6. Findings

This section presents an analysis of the manifestos from the 2011, 2016 and 2020 Irish elections, revealing a growing emphasis on animal protection, particularly farmed animal welfare and wildlife/biodiversity. Left-wing parties generally prioritise animal protection, while right-wing parties focus on its economic implications. Notably, right-wing parties’ attitudes towards animals have evolved positively over the decade. The findings are detailed in three subsections: trends in animal protection salience, analysis of specific issues, and party political analysis.

### 6.1. Trends in Animal Protection Salience

**Figure 1:**
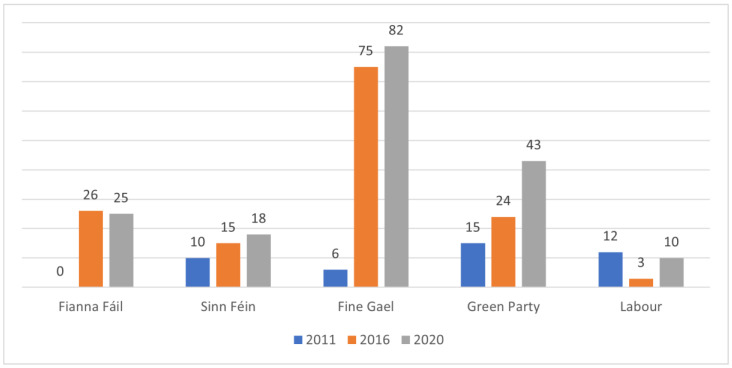
Total number of animal protection statements per political party for Irish national elections in 2011, 2016, and 2020 [34,35,36,37,39,79,80,81,82,83,84,85,86,87,88]. Parties are ranked by number of seats after the 2020 election.

Between 2011 and 2020, animal protection became increasingly prominent in Irish political manifestos, with statements rising from 43 in 2011 to 143 in 2016 and 178 in 2020. Positive statements grew from 29 in 2011 to 74 in 2016 and 113 in 2020. Although the length of party manifestos has varied over the years, the number of statements addressing animal protection has consistently grown. Fianna Fáil was the only party in 2011 without animal protection statements, but all parties included them in 2016 and 2020 (see Figure 1). Anti-animal protection statements varied, comprising 2.3% in 2011, 26.6% in 2016, and 13.5% in 2020. These were primarily linked to Fine Gael and Fianna Fáil’s support for horse and greyhound racing.

**Figure 2:**
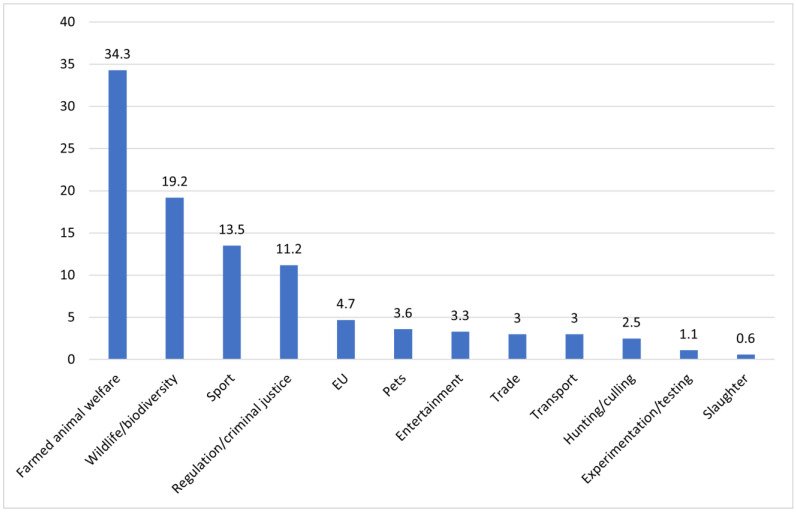
Total number of animal protection statements per issue category for the Irish national elections of 2011, 2016, and 2020 combined.

For the Irish general elections in 2011, 2016, and 2020, farmed animal welfare (34.3%) and wildlife/biodiversity (19.2%) were the most salient issue categories. Furthermore, farmed animal welfare was the most salient issue in each general election, followed by wildlife/biodiversity. Figure 2 illustrates the total number of animal protection statements per issue category for the Irish national elections of 2011, 2016, and 2020 combined.

### 6.2. Analysis of Salience of Animal Protection Issues by Issue

Between 2011 and 2020, the number of farmed animal welfare statements by Irish political parties increased, rising from 11 in 2011 to 64 in 2020. Statements on wildlife and biodiversity also saw a rise, from 8 in 2011 to 32 in 2020.

### 6.3. Party Political Analysis of 2020 Manifestos

This section analyzes the 2020 general election manifestos, summarising key policy issues in Table 3. It then explores animal protection concerns across sectors, including agriculture, aquaculture, farmed animal welfare (see Table 4), wildlife and biodiversity (see Table 5), horse and greyhound racing (see Table 6), and pets.

#### 6.3.1. Agriculture, Aquaculture, and Farm Animal Welfare

In 2020, all political parties committed to fair prices and greater transparency in the food supply chain. Fianna Fáil supported adding value to the dairy sector in line with national food strategies [34] (p. 94), while Fine Gael committed to developing the aquaculture sector and expanding markets for the pig industry [35] (pp. 59,80). A political divide emerged over intensive farming: Sinn Féin opposed it as contrary to Ireland’s interests [37] (p. 32), and the Green Party advocated extensive farming, with an emphasis on animal welfare, recognising animals as sentient beings [36] (pp. 40–41). Fianna Fáil and Fine Gael did not address intensive farming. Labour did not address farmed animal welfare and aquaculture in its manifesto.

#### 6.3.2. Nature Conservation

In Ireland’s 2020 general election, Fianna Fáil proposed a Threat Response Plan for hen harrier conservation and restoring the National Parks and Wildlife Service [34] (p. 95). Fine Gael pledged to appoint education liaison officers and implement a ban on large trawlers within Ireland’s six-mile limit to sustain inshore fisheries [35] (pp. 80,93). Sinn Féin highlighted the impact of super trawlers, advocating for mandatory CCTV on vessels to monitor illegal practices and protect habitats [37] (p. 21). The Green Party focused on supporting the National Pollinator Plan and addressing harmful pesticides [36] (p. 9). Labour pledged to establish maritime conservation zones to protect fish stocks post-Brexit [39] (p. 14).

#### 6.3.3. Sports

In the 2020 election, Fianna Fáil pledged to secure long-term funding for the horse racing sector [34] (p. 96), while Fine Gael committed to developing the industry with Horse Racing Ireland [35] (p. 60). Sinn Féin did not address horse racing, while the Greens and Labour focused on greyhound racing. The Green Party aimed to phase out public funding for greyhound racing [36] (p. 41), and Labour proposed using the betting levy to fund welfare inspectors and address unwanted dogs [39] (p. 38). Fine Gael also committed to fully implementing the Greyhound Racing Act for industry integrity [35] (p. 60).

#### 6.3.4. Pets and Puppy Farming

In their 2020 manifestos, Fianna Fáil and Sinn Féin did not address pet or puppy breeding issues. Fine Gael highlighted past initiatives, including national dog microchipping and new welfare regulations for pet sales [35] (p. 62). Labour proposed stricter enforcement of dog breeding laws, including inspections and penalties [39] (p. 13). The Green Party also called for stricter dog breeding regulations and specified rules on breeding, keeping, and selling animals [36] (p. 41).

## 7. Discussion

### 7.1. Trends of Animal Protection Salience

Between 2011 and 2020, animal protection issues gained prominence in the Irish general election, with increased attention paid to farmed animal welfare and wildlife/biodiversity, consistent with the findings in other European countries such as Belgium and the Netherlands [29]. Fine Gael made the most statements, while the Green Party put forward the most ambitious proposals, including a shift towards extensive livestock farming with a greater emphasis on animal welfare, the recognition of animals as sentient beings, and tighter restrictions on the harmful use of pesticides and insecticides.

Despite the increased focus on animal protection, key issues received limited attention. The puppy trade was absent from party manifestos. Only the Green Party comprehensively addressed animal slaughter, which affected 1.9 million cattle, 3.2 million sheep, and 3.3 million pigs in 2023 [89], and highlighted specific concerns such as badger culling, hunting with hounds, and live hare coursing [36]. The Green Party was the sole political party to address animal testing, a contentious issue in Ireland [90]. Important issues such as chicken lameness, poor fish farming conditions, and the use of sow stalls and farrowing crates are largely overlooked by most parties.

### 7.2. Politically Salient Topics of Animal Protection

#### 7.2.1. Farmed Animal Welfare

The political significance of farmed animal welfare in Ireland has steadily increased from 2011 to 2020, influenced by initiatives like the Food Harvest 2020 programme aimed at expanding the agri-food sector [45]. This programme heightened public discourse on animal welfare alongside consumer and environmental [45]. The Animal Health and Welfare Act 2013 imposed legal duties on animal owners to ensure welfare standards, and the Animal Welfare Strategy 2021–2025, based on One Health and One Welfare principles, further emphasised this interconnectedness [91].

Despite legislative progress, significant challenges remain. In 2023, RTÉ exposed welfare abuses involving male calves, a by-product of the dairy industry, while reports in 2024 revealed poor conditions for pigs [92,93]. Although political parties have proposed various policies, critical issues such as piglet mortality, calf welfare, and the welfare of broiler hens are still inadequately addressed. As Wedderburn [94] noted, addressing these welfare concerns can also improve economic efficiency by reducing health-related losses. These findings are also in line with Vogeler [28], who noted that even countries with advanced policies often fall short in addressing less visible welfare issues.

The Green Party succeeded in banning fur farming through the Animal Health and Welfare and Forestry (Miscellaneous Provisions) Act 2022, a pledge made in its 2020 manifesto [36,95]. While Fianna Fáil and Fine Gael prioritised the economic aspects of agriculture, they largely overlooked welfare concerns. In contrast, the Green Party has consistently championed progressive animal welfare policies, which is in line with broader European trends in Green Party advocacy [25,27,29].

#### 7.2.2. Wildlife/Biodiversity

Although wildlife and biodiversity issues received limited attention in 2011, Irish political parties increased their focus in 2016 and 2020. In 2020, Fianna Fáil, Fine Gael, and the Green Party extended their emphasis from fisheries to include national parks. Fianna Fáil advocated for enhancing biodiversity through a thorough restoration of the National Parks and Wildlife Service [34], while the Green Party called for better resourcing of national parks, related services, and environmental NGOs to fulfil their functions and explore new initiatives [36]. This heightened focus may be attributed to the 2019 UN Convention on Biological Diversity report, which highlighted accelerating habitat loss and species decline [96]. Connaughton [97] noted that Ireland’s biodiversity conservation efforts have been challenged by ongoing disputes between the National Parks and Wildlife Service and private property owners concerning the Birds and Habitats Directives. In terms of protecting endangered species, Irish political parties have prioritised the hen harrier, leading to additional protections under the Forestry Programme 2023–2027, which introduced new measures to safeguard this species beyond designated areas [98].

#### 7.2.3. Horse and Greyhound Racing

A notable shift occurred in the salience of animals in sport, which was absent in 2011. By 2016, horse and greyhound racing was highlighted by centre- and centre-right-wing parties, with Fianna Fáil pledging to maintain Ireland’s global leadership in horse racing [80] (p. 30) and Fine Gael emphasising its importance to rural Ireland [82] (p. 16).

In the 2020 general election, the Green Party critiqued greyhound racing, advocating for an end to public funding. This scrutiny followed a 2014 report by Indecon International Consultants, which exposed financial issues and reputational damage at the Irish greyhound racing board, mainly due to doping scandals [99]. This led to increased political scrutiny, updates to the Welfare of Greyhounds Regulations in 2016, and the enactment of the Greyhound Racing Act 2019 to enhance regulatory oversight.

In 2021, the minister for Agriculture, Food and the Marine, Charlie McConalogue (Fianna Fáil), endorsed the draft Horse and Greyhound Racing Fund Regulations, emphasising the importance of the fund in supporting the economic and social development of both industries [100]. In 2024, the government had allocated EUR 95 million to the fund, of which EUR 76 million was earmarked for horseracing, an increase of EUR 3.2 million from 2023 [69]. Despite this allocation, the government announced an external review to assess the effectiveness of the fund in maintaining high animal welfare standards. The allocation has faced sustained criticism from animal welfare organisations and other stakeholders, who argue that these industries receive a disproportionate amount of taxpayer funding [101]. Documentaries such as The Dark Side of Horse Racing and RTÉ Investigates: Greyhounds Running for Their Lives [19,20] have also highlighted the deep-rooted animal cruelty associated with these sports, making the eradication of such practices a challenging task.

## 8. Conclusions

The analysis of Irish political party manifestos from the 2011, 2016, and 2020 general elections reveals a growing recognition of animal protection as a policy issue. Fianna Fáil and Fine Gael have primarily approached these issues through an economic lens, focusing on the cultural and economic significance of industries like horse racing and live cattle exports. In contrast, Sinn Féin and Labour have given moderate attention to animal protection, while the Green Party has consistently led the call for progressive reforms.

Key developments, such as the Food Harvest 2020 programme, which tied farmed animal welfare to economic and ethical concerns and increased public awareness of biodiversity loss, prompted parties to expand their focus on environmental and wildlife issues. By 2020, party manifestos had evolved from narrow economic concerns, such as the profitability of the fishing industry, to include broader topics like national parks, biodiversity, and wildlife protection. However, significant gaps remain, with critical issues like farm animal mistreatment, the puppy trade, slaughter practices, animal testing, and hunting insufficiently addressed.

This study highlights the growing influence of public awareness of animal sentience and the ethical obligations on political discourse, confirming wider societal shifts in Western society. Future research could explore the relationship between manifesto commitments and subsequent government action, providing insights into how party pledges translate into tangible policy changes. In addition, cross-national comparative studies could deepen our understanding of how political contexts shape the integration of animal welfare into public policy, further linking politics, public attitudes, and evolving ethical commitments to animals.

## Figures and Tables

**Table 1:** Irish political parties that published manifestos for each general election in 2011, 2016, and 2020, their political orientation, and seats won at the 2020 general election.

Political Party	Political Orientation	Seats in 2020 General Election
Fianna Fáil (Soldiers of Destiny)	Centre	38
Sinn Fein (We Ourselves)	Left	37
Fine Gael (Family of the Irish)	Centre-right	35
Green Party (Comhaontas Glas)	Left	12
Labour Party (Páirtí an Lucht Oibre)	Left	6

**Table 2:** Animal protection tags and corresponding issue categories.

Tag	Issue Categories
Entertainment	Circuses, (petting) zoos, dolphinariums, animal fairs.
EU	EU Common Fisheries Policy, EU Pigs Directive, and other EU-related issues.
Experimentation/testing	Animal testing, genetic engineering
Farmed animal welfare	Livestock, fish, fur farming, use of medication, and promotion of meat replacers and plant-based diets.
Hunting and culling	Issues and regulations relating to hare hunting, badger culling, and hunting with hounds.
Pets	Dogs, horses, exotic pets, and related regulations.
Regulation/criminal justice	General animal protection rules, proposed changes to legislation, governance structure, criminal law, food labelling, VAT rates for animal products, animal protection services (e.g., vet, rehoming), sanctions, punishments for animal abuse and neglect, support for animal protection organisations, animal helplines, police, and other services.
Slaughter	Regulations regarding slaughterhouses and non-stun slaughter.
Sport	Horse and greyhound racing: breeding, training, competition, and animal welfare.
Trade	Import/export of animals and animal products, WTO, and related regulation.
Transport	Live animal transport
Wildlife/biodiversity	Fishing industry, wild animals in nature, water and cities, insects, and regulations for biodiversity and animal protection.

**Table 3:** Animal protection policies covered by the Irish political parties in the 2020 general election. Parties are ranked by number of seats after the 2020 election. An ‘x’ indicates that the party included the issue in their manifesto.

Issue Category	Political Party				
	Fianna Fáil	Sinn Fein	Fine Gael	Green Party	Labour Party
Entertainment			x	x	
EU	x	x	x	x	
Experimenting/testing				x	
Farmed animal welfare	x	x	x	x	
Intensive farming		x		x	
Aquaculture	x		x		
Farmer wellbeing	x	x	x		
Economy related to farming	x	x	x	x	
Hunting/culling				x	
Pets			x	x	x
Regulation/criminal justice	x		x	x	x
Slaughter				x	
Sports	x		x	x	x
Horse racing	x		x		x
Greyhound racing			x	x	x
Trade	x	x	x	x	
Transport	x		x	x	
Live export	x		x	x	
Wildlife/biodiversity	x	x	x	x	x
Fishing industry		x	x		x
Protection of wildlife	x	x	x	x	x

**Table 4:** Irish political parties’ manifesto statements related to farmed animal welfare for the 2020 general election.

Political Party	Farmed Animal Welfare Issue			
	Intensive farming	Aquaculture	Farmer wellbeing	Economy related to farming
Fianna Fáil	No policy	‘Review the current implementation of the recommendations of the Report of the Independent Aquaculture Licensing Review Group’.	‘Ensure a fair price for farmers with a EUR 200 per head suckler cow payment scheme’.	‘Support the dairy sector to continue adding value to high quality product in line with national food strategies’.
Sinn Fein	‘Intensive farming models are not in Ireland’s interests’.	No policy	‘The current level of payment to the suckler farmer needs to be enhanced to encourage good husbandry and the proper weaning of calves’.	‘We will also provide for an additional suckler cow scheme to increase the payment to EUR 200 per cow for the first 15 cows in the herd’.
Fine Gael	No policy	‘We are committed to the future development of the aquaculture sector’.	‘We will publish a new animal welfare strategy for Ireland, building on this work, and providing strategic direction for the sector in terms of welfare’.	‘We will work with stakeholders in the pig sector on the successor strategy of Foodwise 2025, open new markets and focus on animal health in the sector’.
Green Party	‘An extensification of the animal agricultural model, one which places emphasis on animal welfare’.	No policy	‘Irish agricultural policy should support farmers to diversify away from an over reliance on dairy and beef production for commodity export markets’	‘Adopt higher welfare standards for all farmed animals and press for measures under the CAP which will support those implementing higher welfare standards’.
Labour Party	No policy	No policy	No policy	No policy

**Table 5:** Irish political parties’ manifesto statements related to wildlife and biodiversity for the 2020 general election.

Political Party	Wildlife/Biodiversity	
	Fishing Industry	Wildlife protection
Fianna Fáil	No policy	‘Ensure the National Parks and Wildlife Service (NPWS) completes a Threat Response Plan for the conservation of Hen Harriers on designated land’.
Sinn Fein	‘We advocate the compulsory installation of CCTV onboard super trawlers to monitor their fishing and processing facilities to stop illegal or unethical practices such as under-reported fishing’.	‘As well as acting as a boundary and serving to enclose livestock in fields, hedgerows act as an important factor in the habitat of various animals and plants’.
Fine Gael	‘We are committed to the sustainable development of the fisheries sector, ensuring that stocks are protected so the next generation have the opportunity to continue the family tradition of deriving an income from the sea’.	‘We will appoint Education Liaison Officers in each of our National Parks to work with schools across the country, in order to promote the importance of biodiversity and the natural world’.
Green Party	No policy	‘Tighten restrictions on the harmful use of pesticides and insecticides’.
Labour Party	‘Labour will set up maritime conservation zones to allow Irish fish stocks to recover and also to protect Irish waters from overfishing post-Brexit’.	

**Table 6:** Irish political parties’ manifesto statements related to sports for the 2020 general election.

Political Party	Sports	
	Horse racing	Greyhound racing
Fianna Fáil	‘Secure the long-term funding of the horse racing sector to ensure its prestige and credibility as the global leader is fully maintained’.	No policy
Sinn Fein	No policy	No policy
Fine Gael	‘We are fully committed to the future of horseracing and will work with Horse Racing Ireland in developing the industry over the coming years’.	‘We will fully implement the Greyhound Racing Act 2019, strengthening integrity in the industry and providing for a new system of traceability’.
Green Party	No policy	‘Phasing out of public funding to the greyhound racing industry’.
Labour Party	Labour will ring-fence part of the Betting Levy to fund animal welfare inspectors to ensure only the highest practice is permitted in any sports or pursuits involving animals (such as greyhound racing and horseracing) and to reform the situation of unwanted dogs’.	

## Data Availability

The original contributions presented in this study are included in the article. Further inquiries can be directed to the corresponding author.
